# Transnational Criminal Enterprise: a Qualitative Social Network Analysis of the Production and Trade in Falsified Medicines

**DOI:** 10.1017/S000397562510009X

**Published:** 2025-12-03

**Authors:** Heather Hamill

**Affiliations:** Department of Sociology, University of Oxford, United Kingdom

**Keywords:** Organised Crime, Transnational Criminal Networks, Falsified Medicines, Trust And Cooperation, Social Network Analysis

## Abstract

This article investigates the organization of a transnational criminal enterprise through a detailed case study of Operation Singapore, a network producing and trafficking falsified pharmaceuticals from China to the United Kingdom. Drawing on law enforcement records, court transcripts, digital communications, and expert interviews, the study maps the structure and strategies of this criminal enterprise. It applies Peter Reuter’s theory of criminal organization, and Niles Breuer and Federico Varese’s typology of network forms to show how functional roles shape organizational structure: production was hierarchical and security-driven, while distribution was decentralized and transactional. Trust was not based on kinship or ethnicity but constructed through moral appeals and restricted information flows. The case reveals how criminal actors exploit legal frameworks and commercial infrastructures to mask illegal activity and blur boundaries between legal and illegal markets. These findings challenge static models of organized crime and call for a more dynamic, relational understanding of criminal enterprise.

## Introduction

CROSS - BORDER CRIME has gained increasing prominence in academic scholarship and policy discourse over the past two decades [Allum and Gilmour 2022; Madsen 2009; Morselli 2009; Rose 2020]. Common manifestations of transnational crime include trafficking in counterfeit, substandard, or illegal products [Transcrime 2016], human trafficking [Campana 2016], and wildlife trafficking [Witbooi *et al*. 2020; Wong 2019].

Within this landscape, the production and distribution of falsified medicines represent a particularly consequential and under-examined dimension of transnational crime. Defined by the World Health Organization (2024) as products that are deliberately and fraudulently misrepresented with regard to their identity, composition, or source, falsified pharmaceuticals pose grave risks to public health and institutional trust [McManus and Naughton 2020]. Patients may suffer directly from toxic ingredients or the absence of therapeutic efficacy, for example, when active ingredients are missing or substituted with incorrect dosages [Newton *et al*. 2008]. Besides individual harm, the proliferation of such medicines contributes to antimicrobial resistance [Cavany *et al*. 2023], prolongs illness, and inflates costs borne by health-care systems [World Health Organization 2024].

The actors involved in these activities often do not conform to traditional organized crime stereotypes, including those belonging to long-standing criminal syndicates. Many participants are entrepreneurs with prior experience in legitimate markets who exploit legal ambiguities, regulatory loopholes, and globalized supply chains to pursue profit. This convergence of legal and illegal economic enterprise makes the study of falsified medicines a fascinating lens through which to examine the organization of illegality, the construction of trust in extra-legal environments, and the permeability of boundaries between formal and informal economies.

This article contributes to the sociological understanding of a transnational criminal enterprise through an empirical analysis of “Operation Singapore”, a multi-jurisdictional investigation into the trafficking of falsified medicines from China into the United Kingdom (UK)^1^. Drawing on a rich and unusually detailed corpus of law enforcement records, judicial transcripts, digital communications, and expert interviews, the study reconstructs the organizational architecture of the network responsible for producing, brokering, and distributing falsified cancer, heart disease, and psychosis medications. These products entered the legitimate UK pharmaceutical supply chain and reached patients, but lacked the required quantity of active ingredients.

The analysis applies Reuter’s [1983] theory of criminal enterprise and Breuer and Varese’s [2023] typology of organized crime networks to the empirical case. This provides a theoretically informed account of how illegal pharmaceutical markets operate in high-income countries.

The study uses qualitative social network analysis to identify the interaction between centralized production hubs and decentralized distribution systems. It highlights how actors exploit regulatory gaps—particularly those in the European Union’s (EU) parallel trade system—to circulate falsified products under the guise of legitimate commerce. Moreover, the analysis sheds light on how trust is manufactured and maintained in illicit markets characterized by high uncertainty and legal risk.

The article is structured as follows. First, it situates the study within existing academic literature on transnational organized crime and falsified medicine supply chains. The methodological section describes the qualitative social network analytical approach employed. The findings section presents a detailed case study, Operation Singapore, focused on investigating networks trafficking falsified medicines from China to the UK. This section describes the key actors, organizational scale, and structure of the supply chain from production to distribution and the investigative processes that led to their exposure. The analytical discussion addresses key themes of network organization, criminal enterprise dynamics, trust and cooperation mechanisms, and regulatory vulnerabilities. The concluding discussion reflects on the theoretical implications of the case, particularly how it sheds light on the shifting boundaries between legal and illegal markets and the challenges that arise within globalized, highly fragmented markets.

### Transnational criminal enterprise

This article examines the organization of transnational criminal enterprise. This phenomenon is historically rooted in trade across territorial boundaries. As soon as formal systems of exchange and transactional norms were established, however loosely conured, efforts commenced to circumvent them. In 1995, the United Nations defined transnational crime as “offenses whose inception, magnitude, and/or repercussions, whether direct or indirect, span multiple nations” [United Nations Office on Drugs and Crime [UNODC], 2002: 4]. Although the original UN classification identified eighteen specific types of transnational crimes, counterfeit goods—including counterfeit pharmaceuticals—were notably absent. Over time, and particularly following the Palermo Convention (2000), the global framework has evolved. International organizations such as the World Customs Organization [WCO 2024] and INTERPOL now explicitly identify counterfeit trafficking as a core element of transnational criminality [INTERPOL 2023].

Operationally, transnational criminal activities are sustained through cooperation among multiple actors, whose coordination and organization constitute a criminal enterprise. The United Nations Convention against Transnational Organized Crime (2000) defines such entities as “a group of three or more persons existing over a period of time acting in concert with the aim of committing crimes for financial or material benefit.” However, this definition offers limited analytical precision: it fails to differentiate organizations based on their size, organizational structure, and internal logic and relationships, whether based on kinship, businesslike coordination, or fluid associations [Levi 1998; von Lampe 2001]. In addition, traditional conceptualizations of organized crime have typically emphasized hierarchical, rigidly structured groups exemplified by the Italian American Mafia [Cressey 1967], the Japanese Yakuza [Hill 2003], the Sicilian Mafia [Gambetta 1996], and Hong Kong Triads [Wang 2017]. These models, however, fail to capture the complexity, flexibility and variability present within illegal production and trade networks.

This study instead adopts a theoretical framework rooted in Peter Reuter’s [1983] critical perspective, which challenged assumptions in academic scholarship about the cohesion and size of criminal organizations. Reuter [1983] and subsequent authors argued that illegal enterprises resembled their legitimate economic counterparts more than is acknowledged [Morselli 2005]. They face similar pressures—market competition, supply chain logistics constraints, and fluctuating consumer demand—but also grapple with challenges unique to the condition of illegality. These include secrecy, absence of legal recourse, and increased transactional risks. Consequently, criminal enterprises tend to remain small in scale, tightly organized, and less capable of growth than their legal counterparts [Bruinsma and Bernasco 2004; Reuter, 1983]. The absence of audited records to verify such businesses’ financial health and operational success further limits investments and growth. Crucially, Reuter highlighted how criminal enterprises exploit regulatory ambiguities and enforcement lacunae. By leveraging legal frameworks—such as parallel trade systems—they obscure their operations behind a veneer of legitimacy, thereby minimizing detection and circumventing sanctions [Lavezzi 2014; Reuter 1983].

This analytical approach is further enriched by typologies developed to differentiate the internal specialization and division of labour in organized criminal activities. Scholars such as Shortland and Varese [2016] distinguish between market-oriented enterprises focused on producing and trading illicit goods, and governance-oriented groups concerned with protection, arbitration, and conflict resolution within illegal markets. Breuer and Varese [2023] expanded on this typology by examining how organizational forms correspond to primary criminal functions—production, trade, or governance. Their comparative analysis of drug distribution networks and governance-focused entities, including the Sicilian Mafia and the Provisional Irish Republican Army (Provisional IRA), revealed that criminal structures are shaped not by culture or territory alone, but also by the functional imperatives of their illegal activities. Production-oriented groups, particularly in counterfeit goods or drug manufacturing, tend to mirror legitimate firms exhibiting characteristics of small-scale industrial or agricultural operations. Technological requirements, capital intensity, and exposure to enforcement dictate their hierarchy, size, and labour practices. By contrast, trade- and transport-focused networks—such as those involved in trafficking drugs, people, or wildlife—exhibit flatter structures and more fluid membership. Their operations span broader geographies and involve a division of labour tailored to cross-border logistics, brokerage, and negotiation.

This article integrates these theoretical perspectives to contribute to a nuanced sociological understanding of transnational criminal enterprise. It foregrounds the structural heterogeneity of illicit networks and emphasizes the need for frameworks that account for the functional diversity within organized crime.

### Trust and cooperation

Trust and cooperation are essential to any economic enterprise, but their importance and form vary between legal and extra-legal contexts. Unlike legal businesses, criminal enterprises cannot rely on third-party enforcement mechanisms such as courts to resolve disputes and ensure compliance [Gambetta 1996; McCarthy *et al*., 1998]. However, the importance and foundations of trust in the extra-legal context remain subject to ongoing debate.

On one side of the argument, authors such as Paoli [2002] suggest that trust is essential in organized crime because it reduces uncertainty, facilitates collaboration, and enhances the longevity of criminal enterprises. Without legal safeguards, criminals must rely on personal relationships, reputation, and informal enforcement mechanisms to ensure reliability among partners. Trust is therefore more likely to be embedded within ethnic, familial, or fraternal structures, which serve as protective barriers against betrayal.

In contrast, Von Lampe and Johansen [2004] take a more skeptical view, arguing that the role of trust in criminal enterprises is overstated in criminological literature. They acknowledge that trust can be a stabilizing factor but contend that it is not a prerequisite for criminal cooperation and is often fragile. Instead, cooperation in loosely connected criminal enterprises can be facilitated by functional alternatives such as limiting information sharing, economic incentives, threats, and violence.

This article contributes to this debate by examining the mechanisms that build and reinforce trust and cooperation within a specific criminal enterprise. The case under examination concerns an enterprise producing and trading illegal goods. It is not a governance-type group, such as a mafia organization, and operates without monopolist territorial or market governance aspirations.

Methodologically, this study takes a qualitative approach to explore Reuter’s hypotheses regarding criminal enterprises and Breuer and Varese’s typology on the network size and structure of groups specializing in the production and trade of illegal goods. The analysis proceeds in three stages. First, it examines the structure and scale of Operation Singapore, hypothesizing that the organization is comparatively smaller and more fragmented than its legal counterparts and that the production network exhibits more hierarchical features than the distribution network. Second, it explores the impact of regulation, positing that specific regulatory frameworks inadvertently created exploitable opportunities that facilitated the group’s operations. Finally, the article investigates how trust-related challenges are navigated among spatially dispersed actors within the network.

### The trade in substandard and falsified medicines

This article focuses on the production and trade of falsified medicines. It is therefore situated within research on the broader issue of substandard and falsified (SF) medicines, which pose a significant threat to global public health [Cockburn *et al*., 2005; Johnston and Holt, 2014]. According to the World Health Organization [WHO 2017], *substandard medicines* are authorized medical products that fail to meet their established quality standards or specifications due to deficiencies, such as inadequate manufacturing processes or degradation caused by improper storage or transportation conditions. This deterioration can substantially diminish the medicine’s therapeutic efficacy. In contrast, *falsi*fi*ed medicines* are deliberately misrepresented regarding their identity, composition, or source. Such medicines frequently lack the requisite active pharmaceutical ingredients (APIs) necessary for their intended therapeutic effect. Additionally, the WHO identifies *unregistered or unlicensed medical products* as those that have not undergone evaluation or received approval from the relevant national or regulatory authorities in the intended market of distribution or use [WHO 2017].

The consequences of poor quality medicines are severe and include treatment failures, prolonged illness, and mortality. These risks are particularly pronounced in low- and middle-income countries (LMICs), in which regulatory oversight is often less rigorous and access to quality pharmaceutical products more limited. The global prevalence of substandard and falsified medical products in LMICs is estimated at slightly over 10% [WHO 2017], while a study in Ghana revealed that approximately 37% of antimalarial medicines sampled from licensed retail outlets were substandard [Wilson *et al*. 2017].

Despite growing international policy attention and regulatory efforts, the empirical study of substandard and falsified medicine production and trade remains limited because of challenging research conditions. The inherently clandestine nature of illegal markets, designed to avoid detection, makes the systematic collection of empirical evidence extremely difficult [Antonopoulos and von Lampe 2016]. The opacity and complexity of global pharmaceutical supply chains involving multiple jurisdictions, regulatory environments, and commercial partners compound the problem [Hamill *et al*. 2019; Pisani *et al*. 2019], alongside the unwillingness or inability of commercial and regulatory stakeholders to disclose operational details. Variability in legal frameworks and enforcement mechanisms between countries further complicates investigative efforts. Determining whether a product is authentic, substandard, falsified, or degraded requires sophisticated laboratory analyses [Roth, Biggs and Bempong 2019], which are often prohibitively expensive for low- and middle-income countries operating under severe resource constraints [Wickett *et al*. 2024]. These detection difficulties have led to variable prevalence rates of substandard and falsified medicines across countries, further impeding comparative research [Hamill *et al*. 2021]. Finally, the legitimate pharmaceutical sector is constantly in flux, with innovation and rapid product turnover driving continual change in the substandard and falsified medicines market and exacerbating the challenges affecting systematic research.

An important exception is the study by Hall *et al*. [2017], which examined the structure and functioning of the illegal medicine market in the UK and the Netherlands. Their research highlights a fluid and adaptable market characterized by porous boundaries between legal and illegal activities and between online and offline marketplaces. Participants ranged from small-scale individual actors to more organized criminal networks, suggesting that the trade is shaped less by monopolistic criminal syndicates and more by opportunistic criminal entrepreneurship operating in hybrid spaces. The authors, however, were not able to access detailed information about the production of these medicines.

Building upon these findings, the present research offers a detailed case study of a criminal production and trade enterprise operating in China, Belgium, and the UK. The qualitative analysis seeks to illuminate the mechanisms through which such enterprises are organized and sustained, contributing to a more nuanced sociological understanding of the falsified medicine trade.

## Methods and data

### Methodological approach

This study employs a qualitative social network analysis based on a detailed case study of what constitutes the most severe documented breach of the UK’s regulated pharmaceutical supply chain. Between December 2006 and May 2007, significant quantities of falsified prescription medicines—specifically Casodex (for cancer treatment), Plavix (for heart disease), and Zyprexa (for psychosis)—were illicitly introduced into the UK’s wholesale pharmaceutical market. The medicines were fraudulently presented as legitimate products for sale in France. Subsequent investigations established that they had been manufactured in China and routed through Hong Kong SAR, Singapore, and Belgium before entering the UK. Upon arrival, the products were repackaged and sold to licensed wholesalers who, unaware of their origin, distributed them to hospitals, clinics, and care homes. Approximately 72,000 packs—equivalent to 2.1 million doses, with an estimated retail value of £7 million—penetrated the legitimate pharmaceutical supply chain.

Drawing upon the UK Medicines and Healthcare Products Regulatory Agency’s (MHRA) extensive investigation—Operation Singapore—this study reconstructs the transnational criminal networks producing and distributing these falsified medicines. The analysis spans six countries and is grounded in primary documentary evidence, judicial records, and supplementary interviews.

Studying transnational criminal enterprises poses significant methodological challenges [Hosford *et al*. 2022]. As early as the 1960s, Donald Cressey [1967] identified a number of core difficulties in researching covert criminal organizations, including participant secrecy, investigative confidentiality, and the interpretive biases of informants and investigators. These challenges remain salient, particularly in the context of the falsified medicines trade, in which prosecutions are rare and data access is highly restricted. Consequently, this study adopts a single-case research design. Although such an approach limits opportunities for empirical generalization, it permits the in-depth examination of complex and concealed organizational structures and supports theoretical refinement [Small 2009].

### Data sources

The empirical foundation for this research consists of 166 pages of investigative documents compiled over four years during Operation Singapore. These include materials from parallel legal proceedings in France and the United States. The author obtained direct access to documentation integral to the MHRA’s investigation and court-admitted evidence from the UK trials of individuals implicated in the case.

Central to the data is a 22-page chat log recovered from the personal computer of the Chinese manufacturer responsible for producing the falsified medicines. This document contains verbatim transcripts of communications and negotiations between the Chinese supplier and a European intermediary between July 3 and July 24, 2007. In addition, extensive trial evidence details each defendant’s role within the wider criminal network.

Further quantitative data were drawn from MHRA-led product recall procedures, including shipping records, importation dates, product descriptions, and batch quantities. These documents proved instrumental in reconstructing the logistical and operational dimensions of the supply chain and allowed for triangulation of key findings across multiple data sources.

Between 2023 and 2024, the author conducted multiple informal interviews with the MHRA’s chief investigator assigned to Operation Singapore. These interviews clarified timelines, investigative procedures, and international coordination, and provided contextual insights into the principal actors that are not evident in the formal documentation.

Parallel investigations in Switzerland and France targeted two individuals identified as intermediaries linking the Chinese manufacturer and the UK-based importer. Publicly available press reports from their court trials provided details of their activities and subsequent convictions.

Supplementary material was drawn from the publicly broadcast documentary *Dan Rather Reports:* “*The Mysterious Case of Kevin Xu*,” aired in the United States on September 13, 2010 (last accessed 24/05/2024). The program documents an investigation led by US Immigration and Customs Enforcement (US ICE) that culminated in the arrest of Kevin Xu, a Chinese national implicated in attempts to traffic falsified pharmaceuticals into the United States and identified by Operation Singapore as the lead manufacturer responsible for the UK imports. The documentary and its transcript include detailed discussions between Xu and US ICE agents, offering a rare first-person account of illicit pharmaceutical production practices.

Given that much of the information analyzed in this study is already publicly accessible—through media reporting, trial documentation, and the documentary—the persons discussed are not anonymized.

### Data analysis

Data were analyzed using a qualitative framework guided by principles of social network analysis, Reuter’s theory of criminal enterprise, and Breuer and Varese’s typology linking network structures to criminal specialization. The analysis proceeded in three stages.

First, a descriptive coding process identified and classified each actor’s operational role within the criminal enterprise, differentiating between production-related functions (for example, manufacturing and logistics) and trade-related functions (such as brokerage, financing, and wholesale distribution). This resulted in a map of the network structures.

Second, a thematic analysis identified recurrent patterns and mechanisms within the network. Key themes included the nature of transnational criminal enterprise interpersonal ties (transactional versus kinship-based), the processes of trust formation and maintenance, security protocols, strategies for evading regulatory scrutiny, and patterns of communication and coordination. This analysis stage also explored actors’ adherence to—or manipulation of—existing regulatory frameworks.

Finally, the synthesis of descriptive and thematic analysis yielded a comprehensive analytical narrative that reconstructs the criminal production and trade network at the heart of Operation Singapore. The result is a sociological account of how illicit pharmaceutical supply chains are organized and sustained across borders, offering insights into the broader dynamics of transnational criminal entrepreneurship.

## Operation Singapore: Production and trade in falsified medicines

### Key actors

The central figures implicated in Operation Singapore formed a loosely coordinated transnational network operating across six countries. At the core was Kevin Xu, a Chinese businessman convicted in the United States in August 2008 and sentenced to seventy-eight months’ imprisonment for distributing counterfeit and misbranded pharmaceuticals. His arrest followed a joint undercover operation conducted by US ICE and the US Food and Drug Administration’s Office of Criminal Investigation (US FDA). Three individuals were arrested in China for suspected involvement in the manufacturing process, but they were released without charge and their precise connection to Xu remains unverified.

In April 2011, UK authorities sentenced Peter Gillespie, a 64-year-old businessman, to eight years’ imprisonment for conspiracy to defraud pharmaceutical wholesalers, pharmacists, and consumers. He was also convicted of selling pharmaceuticals without marketing authorization between January 2006 and June 2007 and of acting as a company director while disqualified. Four of his associates—Richard Kemp, Ian Harding, James Quinn, and his brother Ian Gillespie—were tried but acquitted.

Additional prosecutions occurred in France. In 2017, a Marseille court sentenced Arnaud Bellavoine and Catherine Koubi to five and three years’ imprisonment, respectively, for offenses related to the importation of falsified medicines from China into Europe. Bellavoine, arrested in Spain in October 2012, operated a Mauritius-based offshore company; Koubi ran a brokerage firm in Nice. A warrant was also issued for Arnaud’s father, Bernard Bellavoine, a Tunisian businessman, though he was never apprehended.

The UK MHRA identified these nine individuals as the primary actors in Operation Singapore.

### Products and discovery

Between November 2006 and May 2007, more than 72,000 packs of falsified prescription medicines—comprising over one million tablets with an estimated retail value exceeding £7 million—were illicitly imported into the UK. These included three high-value products: Casodex (cancer treatment), Plavix (heart disease), and Zyprexa (psychosis). Though visually indistinguishable from their authentic counterparts, laboratory analysis revealed severe deficiencies in active pharmaceutical ingredients (API): Casodex and Plavix contained 70–80% of the requisite API, while Zyprexa ranged between 58% and 81%. In some instances, tablets were reportedly composed entirely of sugar [Leroux 2017^2^]. An estimated 32,000 packs—equivalent to 900,000 doses—were dispensed to patients via UK National Health Service (NHS)-approved pharmacies.

No severe adverse reactions or deaths were directly attributed to the falsified medications. Identifying a causal relationship between falsified medicines and patient outcomes is challenging, given that deterioration in patient health could easily be attributed to existing medical conditions. Despite the lack of confirmed harm, regulatory agencies classified the incident as a critical breach of pharmaceutical integrity.

Discovery occurred fortuitously. Suspicions were aroused first when a UK repackaging facility employee observed reversed batch number imprints on Zyprexa blister packs. Separately, a patient complaint concerning easily removed tablet markings prompted Eli Lilly—the nominal manufacturer—to contact the MHRA. Eli Lilly had been collaborating with US authorities to investigate Kevin Xu, who had attempted to introduce similar falsified medicines into the US market. Following Xu’s arrest in Houston, Texas, forensic analysis of his laptop revealed digital evidence linking him to falsified batches of Casodex, Plavix, and Zyprexa. This was then shared with the UK MHRA.

### Production

The falsified medicines originated in Tianjin, a major port city in northern China and a key node in the global pharmaceutical export industry. Kevin Xu, who supervised the manufacturing, claimed to operate a number of legitimate pharmaceutical businesses producing generic medicines for domestic hospitals, boasting: “We are Chinese government supplier actually” [Dan Rather Reports, 2010: 14.57 min]. In 2007, he invited Arnaud Bellavoine to inspect his facilities and referenced substantial investments in laboratory infrastructure: “You can see how we work, and you should know how big money we put into our labs” (Chat log: KX to AB, July 16, 2007).

During interactions with undercover US ICE agents, Xu listed twenty-nine medicines he was capable of falsifying, covering a broad therapeutic spectrum, including treatments for Alzheimer’s disease, ulcers, cancer, cardiovascular diseases, and erectile dysfunction (Viagra). He claimed a production capacity of over 200,000 boxes (equivalent to more than one million individual doses) within a five-day cycle, none of them containing the correct dosage of API [Dan Rather Reports 2010: 13.59–14.30 mins].

### Transportation and distribution

The falsified medicines were smuggled out of China under the false declaration of “Lipoic Acid,” a dietary supplement. Xu controlled two freight companies—Pacific Oriental International and Bestway Nanjing—and admitted to bribing customs officials to ensure unimpeded export: “We have strong relations with Customs. We pay money to Customs” [Dan Rather Reports 2010: 12.45–12.52 mins]. The products were routed from China through Singapore because, according to Xu, “it’s safer than from China to the UK” because “some Asian countries… maybe Malaysia or Singapore has very good relation with the European countries” [*Ibid*.: 18.18–18.34 mins]. In Singapore, the products were repackaged and airfreighted to Belgium under the direction of two Mauritius-based shell companies managed by Bellavoine.

Shipments were fragmented into smaller consignments to mitigate the risk of large-scale interception. According to Xu, “Safe quantity at one time is… 500 strips” [Dan Rather Reports, 13.14 mins]. Once cleared through Belgian customs, Peter Gillespie retrieved the products and transported them to the UK. They were repackaged again before entering the legitimate pharmaceutical distribution network and ultimately reached patients through NHS prescriptions.

### Exploiting regulatory frameworks: Parallel trade

A critical enabler of the operation was the exploitation of European Union (EU) regulations (Regulation (EC) No 726/2004) governing parallel pharmaceutical trade [European Parliament and Council, 2004]. These rules permit the cross-border sale and distribution of pharmaceuticals within the EU, provided that imported products are chemically identical and manufactured by the same producer. The falsified medicines were falsely presented as destined for the French market, which enabled legal entry into the UK under the guise of authorized parallel trade. Although the initial packaging conformed to French specifications, further modifications were made in the UK to reinforce their appearance of authenticity.

### Repackaging and labelling: Signaling authenticity

A significant factor in the success of this enterprise was Kevin Xu’s ability to replicate the packaging of genuine medicines, which he did with considerable attention to detail. He told the ICE agents: “We use a laser machine to print out because we know how Sanofi-Aventis do the drugs, so we do the copy. We use the same machinery” [Dan Rather Reports 2010: 16.09–16.16 mins]. When the medicines arrived at Gillespie’s facility in Basingstoke, they bore three key indicators of authenticity: original manufacturer trademarks (AstraZeneca, Eli Lilly, Sanofi-Aventis), genuine-looking French-language patient information leaflets, and corrected outer packaging that matched legitimate French products. One initially omitted element—the French vignette, an adhesive label required for health insurance reimbursement—was counterfeited by Gillespie and affixed to the products, completing the illusion of authenticity.

### Financial transactions

**Figure 1:**
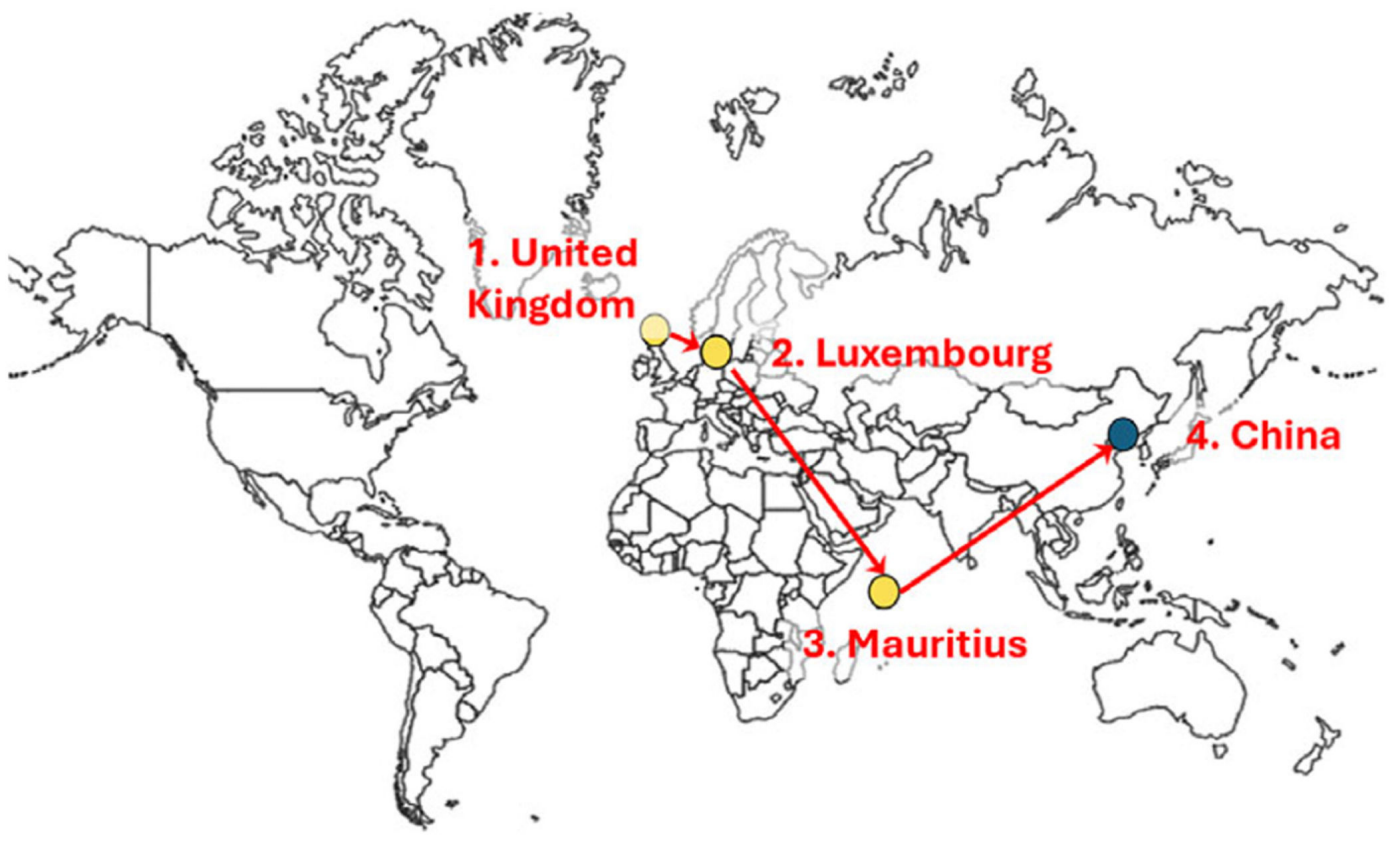
The Money Flow

While direct financial data remain limited, the available evidence points to three key entities facilitating transactions: Kirchberg Handels (Luxembourg) and two Mauritius-based companies, Multiscope Trading and Ridus Trading. As director of Kirchberg Handels, Gillespie managed product sales and profits were channeled through Maghreb Pharma, a Tunisian firm owned by Bernard and Arnaud Bellavoine. Gillespie maintained long-standing financial ties to Maghreb Pharma and conducted business with CMS, his UK-based company. Analysis of payment flows (see Figure 1) suggests that revenue from UK sales was routed through Bellavoine’s offshore companies before reaching the Chinese supplier.

## The organization of a criminal enterprise

### Profit orientation and entrepreneurial motivations

The pharmaceutical sector is one of the most lucrative global markets, with annual expenditures reaching the trillions. In 2021, for example, the UK NHS spent approximately £39.6 billion on pharmaceuticals alone [ONS 2023]. While legitimate corporate actors predominate in this sector, its high profitability and complex regulatory environment make it particularly vulnerable to criminal exploitation.

The primary figures implicated in Operation Singapore—Peter Gillespie, Kevin Xu, and Arnaud Bellavoine—were experienced businesspeople with prior involvement in the legal pharmaceutical industry. The US ICE agent described Xu as “the perfect example of a businessman well-versed and knowledgeable about the products that he offered as well as the benefits and the routes to best introduce them into the commerce of that applicable country” [Dan Rather Reports 2010: 5.37–5.43 mins]. These principal actors capitalized on the persistent demand for life-saving medications to engage in the production and distribution of falsified medicines. Their activities were profit-driven, shaped by rational economic calculation. During judicial proceedings in Marseille, Bellavoine openly acknowledged the instrumental logic underpinning his actions: “I was hungry for money. I had big financial problems… I had the idea of Plavix and Zyprexa. They were very fashionable products, and large volumes were prescribed throughout Europe” [quoted in Deumier and Vergnenègre, 2017^3^].

Despite the illicit nature of their actions, the operational logic of the network mirrored that of legitimate commercial enterprises, consistent with Reuter’s [1983] depiction of rational criminal entrepreneurship. Available estimates suggest that active pharmaceutical ingredients (APIs) account for 40–60% of total production costs in manufacturing legitimate medicines [Hill *et al*., 2018]. Operation Singapore’s profit margins were maximized by significantly reducing the quantity—and thus the cost—of APIs; falsified products such as Zyprexa, Plavix, and Casodex contained amounts far below the required therapeutic thresholds.

A central challenge for transnational criminal enterprises is the covert movement of capital across jurisdictions. Consistent with established laundering strategies [Levi, 2015; Levi and Soudijn, 2020], Gillespie and the Bellavoines channeled illicit proceeds through a network of shell and front companies in Luxembourg and Mauritius. A brokerage firm, Keren SA—directed by Catherine Koubi and based in Nice—was also utilized to coordinate the European distribution of falsified pharmaceuticals, facilitating cross-border financial and logistical flows while maintaining the appearance of legitimacy.

### Structural composition of the network

**Figure 2:**
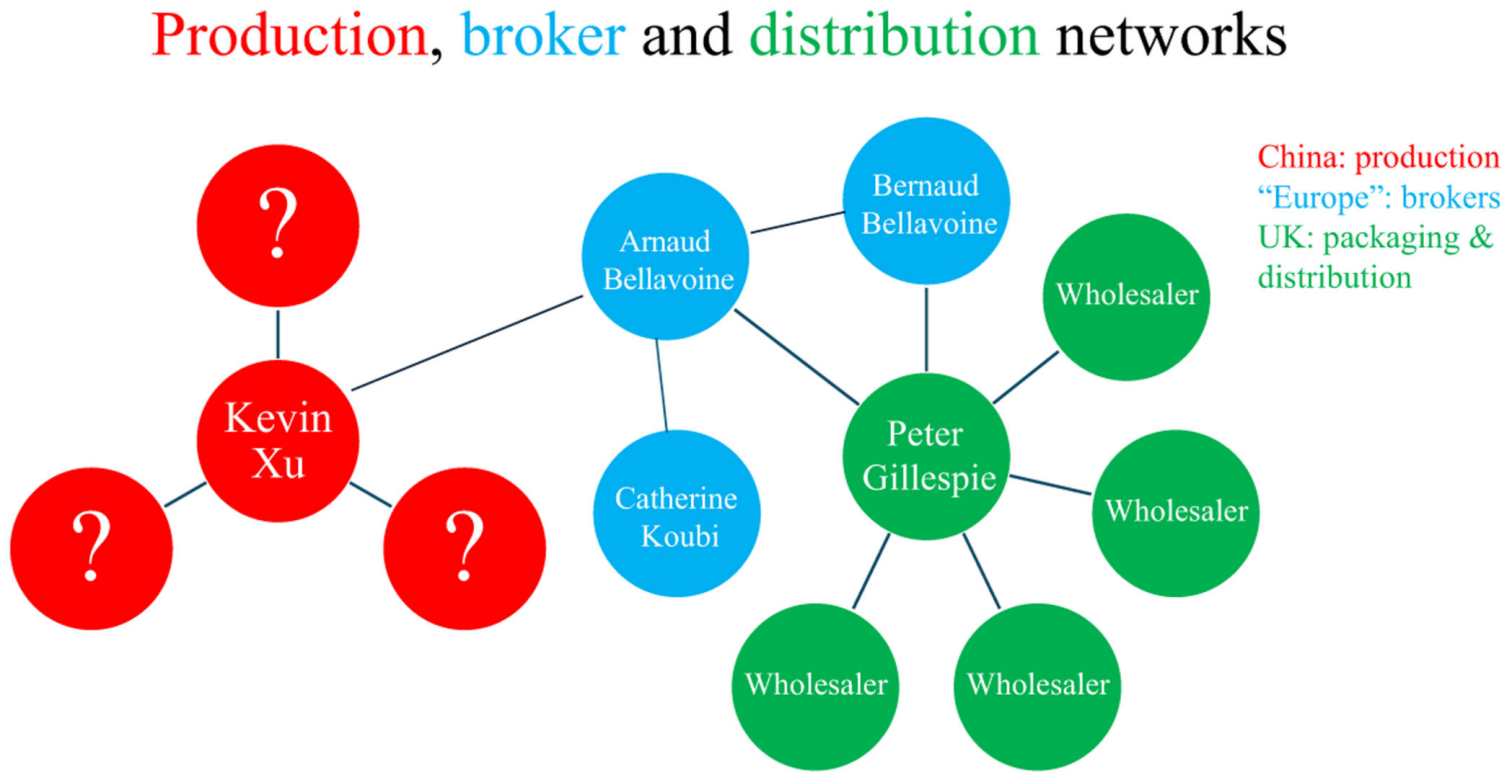
Production, broker and distribution networks Note: In Figure 2, lines indicate confirmed relationships between actors.

Operation Singapore reveals the distinct structural segmentation of transnational criminal networks operating across the production, brokerage, and distribution domains. Based on investigative data (and visualized in Figure 2), the network exhibits hierarchical and transactional elements corresponding to its functional specialization.

Production was centralized in Tianjin, China, under the supervision of Kevin Xu. The production network was small and embedded within legitimate pharmaceutical infrastructure, enabling a dual-use model of licit and illicit output. In a chat-log conversation with Arnaud Bellavoine, Xu claimed that only five people knew about this side of his business: “for this business with you, only one engineer and me and three logistics know” (Chat log KX to AB, July 14, 2007). This structure is in line with Breuer and Varese’s [2023] typology of small, tightly controlled production networks within criminal enterprises.

Arnaud Bellavoine was the critical intermediary between the Chinese production site and the European trade network. He maintained close ties within Europe with his father, Bernard Bellavoine, and Koubi. Peter Gillespie, based in the UK, was the primary recipient and redistributor of the products. His connections to other UK-based associates—who were later acquitted—were reportedly infrequent and operationally compartmentalized.

The European network thus assumed a non-hierarchical, transactional form, consistent with Morselli and Roy’s [2008] framework and Paoli’s [2002] conceptualization of trade-oriented criminal collaborations. Rather than operating as a stable organizational hierarchy, the network functioned through temporary, project-specific partnerships, resembling the fluidity and complexity of legitimate pharmaceutical supply chains but on a reduced scale.

### Trust, secrecy, and security

Trust emerged in this criminal enterprise in two key functions: ensuring compliance between actors and reducing the risk of detection. Trust was established and maintained by mechanisms that were both relational and structural.

### Relational mechanisms

The relationships in this network were primarily transactional, with only one confirmed kinship link, between Arnaud and Bernard Bellavoine. Prior business relations between Bernard Bellavoine and Gillespie likely facilitated the initial contact with Arnaud and established a minimal trust threshold necessary for collaboration.

The most crucial relational axis—that between Xu and Arnaud Bellavoine—remains opaque in origin. Their correspondence, preserved in chat logs, primarily contains discussions of product specifications, pricing structures, and logistical timelines for production and delivery. They had no formal enforcement mechanism available to them should either party breach their agreement, and the chat log reveals efforts to invoke moral obligations and mutual loyalty as trust-building strategies: “for this special business, we need both side mutual support and understand…” (Chat log KX to AB, July 16, 2007). Xu, bearing the initial manufacturing costs, frequently used kinship metaphors—referring to Bellavoine as “brother”— and emphasized exclusivity and mutual dependence in their partnership: “we trust you, I am interested in cooperating with you and do EU market only through you” (Chat log KX to AB, July 16, 2007). Bellavoine reciprocated these trust sentiments: “I respect u and trust u 10000%” (Chat log AB to KX, July 16, 2007). Such statements exemplify trust contingent on immediate mutual interest and pragmatically enforced reciprocal moral obligations [Haas 2016; Hamill *et al*. 2019; Torsello 2008].

### Structural mechanisms

The need to maintain security and secrecy within illicit enterprises is closely linked to trust. Security protocols were deliberately embedded within the structure of the network’s operations. Xu compartmentalized production knowledge to a minimal number of trusted individuals: “for this business with you… only engineer and me know exact products, logistics don’t know exact products” (Chat log KX to AB, July 14, 2007) and misrepresented the product as “Lipoic Acid” in customs declarations. He explicitly stated that logistics personnel were unaware of the actual contents of shipments: “Logistics only know products’ name on paper, they don’t know products in drum. So, for us everything is ok. Now, you need to take care on your side. Now for me everything is clean” (Chat log KX to AB, July 14, 2007). Bellavoine, for his part, reduced his personnel by 60%, a decision Xu praised as a necessary step, saying, “I think you fire people is right, your circle is clean” (Chat log KX to AB, July 14, 2007).

### The absence of violence

In many organized crime networks, violence functions as a tool of control and enforcement. It is used for retaliation, score-settling, extortion, reputational signaling, ensuring compliance, or resolving disputes arising from failed transactions [Gambetta 1996, 2011; Jacobs and Wright 2006; Niezink and Campana 2023]. Operation Singapore displayed no evidence of coercion or physical threat, however. The absence of violence can be interpreted as a strategic choice, as it is often counterproductive in high-value, low-visibility white-collar crime, attracting regulatory scrutiny and increasing enforcement risk [Gambetta 2011].

Compliance and cooperation were achieved through relational and structural mechanisms rather than the threat or use of violence. Mutual dependency, moral framing, and exclusive collaboration were invoked to establish trust, which was maintained through ongoing communication and reaffirmation of shared goals rather than costly signaling [Gambetta 2011; Hamill 2011] or coercive practices such as hostage-taking [Campana and Varese 2013].

### Perceptions and calculations of risk

Understanding criminal decision-making requires attention to actors’ perceptions of detection probability and regulatory enforcement. In this case, both Xu and Arnaud Bellavoine demonstrated an awareness of the risks involved. They adopted precautionary measures—limiting information flows, monitoring regulatory activity, and fragmenting shipments—indicating a strategic approach to risk minimization.

One of the riskiest aspects of criminal enterprise is the establishment of new business relationships, particularly when they involve unfamiliar markets or actors. This vulnerability is starkly illustrated in the events leading to the arrest of Kevin Xu. In footage from the US documentary covering his case, Xu speaks candidly with undercover agents from US ICE, disclosing sensitive operational details about his pharmaceutical falsification business. While his openness may appear reckless, it was the product of a carefully staged deception. ICE agents had arranged a meeting in Bangkok under the guise of representing a pharmaceutical distribution company capable of supplying the necessary documentation to import falsified medicines into the United States. Following this meeting, communications continued over several months, during which the agents placed substantial orders for counterfeit products, amounting to over $167,000. Enticed by the prospect of scaling his business into the lucrative US market, Xu agreed to travel to Houston, Texas, where ICE agents had set up a staged office to simulate a legitimate commercial operation. The footage of his arrest captures Xu’s visible surprise at being deceived [[Dan Rather Reports 2010: 25.08–25.20 mins]. His willingness to engage so openly with new partners may reflect the economic incentives at stake and a miscalculation of the enforcement risk in an unfamiliar jurisdiction. It is plausible that, had the same overtures come from European officials—where Xu was already operating—he would have exercised greater caution. This misjudgment proved decisive, leading to his arrest and the exposure of the broader transnational network to which he was central.

### Intersections between legal and illegal supply chains

A defining characteristic of this enterprise was its ability to straddle legal and illegal pharmaceutical supply chains. For illegal drugs such as heroin, both the raw materials and final products are prohibited in many countries. In this case, although the products were falsified, the ingredients used were not illegal in origin and may have been acquired through legitimate suppliers. Moreover, the movement of products occurred primarily via commercial channels rather than clandestine smuggling routes.

From China to Belgium and eventually to the UK, the goods were interspersed with—and ultimately absorbed into—legitimate distribution systems. This included licensed UK wholesalers, some of whom were unaware of the illegality of the products. The complexity and global scale of contemporary pharmaceutical logistics, combined with the visual indistinguishability of the products, facilitated their integration into hospital and pharmacy supply chains.

The individuals involved were not traditional organized crime figures but businesspeople with prior pharmaceutical sector experience. Unlike participants in tobacco or narcotics smuggling networks [Antonopoulos 2008; Feltran 2019], these actors appeared to specialize in falsified medicines, and there is no evidence of any attempts to diversify into other illegal products. This may reflect the comparatively high-profit margins and the lower risk of detection and prosecution associated with falsified medicines.

### Regulatory exploitation

Central to the enterprise’s success was the strategic exploitation of European Union (EU) and European Economic Area (EEA) parallel trading regulations, which permit intra-regional trade of pharmaceutical products under specific conditions. Once the products cleared Belgian customs, they moved legally across borders to the UK under the guise of French-market products. In the UK, any packaging peculiarities or irregularities could be explained away as being due to the French packaging specifications.

Financial concealment was similarly sophisticated. Shell companies, nominee directors, and inter-jurisdictional transfers allowed the network to move funds with minimal scrutiny. These practices exemplify Reuter’s [1983] argument that economic criminals are adept at exploiting the frameworks—in this case, trade liberalization and regulatory harmonization—designed to facilitate legitimate commerce.

## Conclusions

This article has examined the organization of transnational criminal enterprise through the empirical lens of falsified pharmaceutical production and trade, demonstrating how illicit markets are embedded within—and parasitic upon—legal infrastructures. Operation Singapore offers a striking example of how criminal entrepreneurs take advantage of regulations, fragmented global supply chains, and the very systems designed for legitimate trade. This enterprise operated with a logic that closely mirrors legal businesses, adapting to constraints, responding to incentives, and organizing itself in ways that reflect the demands of the illegal market. Instead of existing on the margins of the economy, this kind of illegal activity often mirrors the same patterns sociologists see in legitimate markets, such as specialized roles, adaptable structures, and a pragmatic response to shifting conditions.

The analysis reinforces Reuter’s [1983] foundational claim that criminal enterprises tend to be small-scale, risk-averse, and structurally constrained by the condition of illegality. The limited size, functional specialization, and absence of durable hierarchies in this case reflect precisely the organizational logic that Reuter described as shaped not by cultural traditions or subcultural identities but by the challenges of coordination, secrecy, and enforcement in the absence of legal protections. Moreover, the structural segmentation between a hierarchical production network and a fluid, transaction-based distribution network substantiates Breuer and Varese’s [2023] typology of organized crime forms. Their argument that the shape of an organization depends on what it primarily does is supported by the clear differences between how the production side and the trade side of the network were structured and operated.

The study also refines our sociological understanding of trust in illegal markets. Trust was not built on shared ethnicity or family ties, as is often assumed in organized crime research. It was earned and maintained through mutual dependence, rhetorical appeals to loyalty, and careful control over who knew what. These are mechanisms that echo Gambetta’s [1996] argument that under conditions of uncertainty, when actors’ trustworthiness is an unknown property, trust must be actively constructed to ensure cooperation without legal enforcement. However, unlike in mafiatype organizations, in which trust is often embedded in long-term relationships and reinforced by violence, the case examined here illustrates a more instrumental and situational form of trust adapted to a market-oriented, task-specific enterprise. Rather than reflecting a criminal code or a cultural bond, the dynamics suggest that trust formation and maintenance in this illegal network is less about cohesion and more about managing uncertainty and avoiding detection. As such, it was a practical and fragile arrangement, shaped by each actor’s position and exposure to risk.

Secrecy was closely entwined with this mode of trust and embedded in the network through strategic compartmentalization, limiting personnel involvement, and the use of deliberate misinformation. Sensitive knowledge—such as the true nature of the products, their destinations, or the broader scope of the enterprise—was confined to a small core of actors. Logistics staff, for example, were unaware of the actual contents of shipments, which were falsely declared to be dietary supplements. Communication between key actors emphasized discretion, and roles were narrowly defined with no unnecessary overlap or information sharing. This strategic segmentation minimized the risk of exposure and enhanced operational security. In this way, secrecy operated not as a secondary feature of criminality but as a fundamental organizational logic that illustrates how illegal enterprises structurally adapt to the demands of concealment within extra-legal environments.

Finally, this case vividly reveals the blurred boundary between legal and illegal markets. Operation Singapore did not operate in isolation from the formal, legal pharmaceutical market but was deeply entangled with it. The actors exploited regulatory loopholes, navigated formal trade rules, and leveraged legitimate commercial infrastructure to conceal the falsified pharmaceuticals behind a façade of legal activity. In this context, illegality appears not as a parallel system but as an alternative mode of participating in formal economic processes by adapting to ambiguity, exploiting gaps, and mimicking legitimacy. The structure of such enterprises reflects the functional demands of their activities, and their operations are often deeply entangled with the economic and regulatory systems of global capitalism.

### Limitations

This study provides a rare and detailed look into a transnational criminal network, but several important limitations must be acknowledged. First, the analysis focuses on a single case, Operation Singapore. Although this case is unusually well documented, it reflects a particular moment in time, a specific set of actors, and a unique regulatory environment. As such, the findings should be seen as illustrative and not representative of all forms of transnational criminal enterprise.

Second, much material comes from official sources, namely court documents and files from the regulatory authority. These sources are rich in operational detail but are shaped by institutional priorities. They tend to spotlight what is legally or procedurally significant, which means that other aspects—such as informal relationships, personal motivations, or behind-the-scenes decision-making—can be harder to access. The chat logs and expert interviews partially fill this gap, but it was not possible to interview the key actors, and that limits how fully we can understand their perspectives.

Finally, while the method used here—qualitative social network analysis—works well for mapping relationships and understanding organizational structure, it cannot fully capture the emotional, symbolic, or moral dimensions of participating in illegal activity. These aspects matter, especially when it comes to understanding how people justify or make sense of their roles within such networks.

These limitations do not undermine the study’s contribution but they do highlight some of the challenges of studying hidden, extra-legal worlds. The very things that make this network interesting—its secrecy, adaptability, and use of legal structures—also make it difficult to see and understand fully. The findings should therefore be read as one window into how a criminal enterprise can operate within and alongside legal markets rather than as a definitive account of all such activity.

## Footnotes

1 This research was funded in whole, or in part, by the Wellcome Trust [222506/Z/21/Z]. For the purpose of Open Access, the author has applied a CC BY public copyright licence to any Author accepted manuscript version arising from this submission.

2 Leroux Luc, 2017. “A Network of Fake Medicines before the Criminal Court of Marseille,” *Le Monde*, March 20, 2017 [https://www.lemonde.fr/sante/article/2017/03/20/un-reseau-de-faux-medicaments-devant-letribunal-correctionnel-de-marseille_5097282_1651302.html, accessed May 26, 2024].

3 DEUMIER Nathalie and Annie VERGNENÈGRE, 2017. “Marseille: 5 Years in Prison for the Organizer of a Trafficking of Falsified Medicines,” *Franceinfo*, May 4, 2017 [https://france3-regions.francetvinfo.fr/provence-alpescote-d-azur/bouches-du-rhone/metropole-aixmarseille/marseille/marseille-5-ans-prisonferme-organisateur-trafic-faux-medicaments-1228083.html, accessed May 26, 2024].
